# Recombinant proteins of *Plasmodium malariae* merozoite surface protein 1 (PmMSP1): Testing immunogenicity in the BALB/c model and potential use as diagnostic tool

**DOI:** 10.1371/journal.pone.0219629

**Published:** 2019-07-25

**Authors:** Yelina B. Elizardez, Wesley L. Fotoran, Andrés J. Galisteo Junior, Izilda Curado, Norival Kesper Junior, Eliana F. Monteiro, Irineu Romero Neto, Gerhard Wunderlich, Karin Kirchgatter

**Affiliations:** 1 Núcleo de Estudos em Malária, Superintendência de Controle de Endemias/Instituto de Medicina Tropical, Universidade de São Paulo, São Paulo, Brazil; 2 Departamento de Parasitologia, Instituto de Ciências Biomédicas, Universidade de São Paulo, São Paulo, Brazil; 3 Laboratório de Protozoologia, Instituto de Medicina Tropical, Universidade de São Paulo, São Paulo, Brazil; 4 Laboratório de Imunoepidemiologia, Superintendência de Controle de Endemias, São Paulo, Brazil; Ehime Daigaku, JAPAN

## Abstract

**Background:**

*Plasmodium malariae* is the third most prevalent human malaria-causing species and has a patchy, but ample distribution in the world. Humans can host the parasite for years without presenting significant symptoms, turning its diagnosis and control into a difficult task. Here, we investigated the immunogenicity of recombinant proteins of *P*. *malariae* MSP1.

**Methods:**

Five regions of PmMSP1 were expressed in *Escherichia coli* as GST-fusion proteins and immunized in BALB/c mice. The specificity, subtyping, and affinity of raised antibodies were evaluated by enzyme-linked immunosorbent assays. Cellular immune responses were analyzed by lymphoproliferation assays and cytokine levels produced by splenocytes were detected by cytometry.

**Results:**

We found that N-terminal, central regions, and PmMSP1_19_ are strongly immunogenic in mice. After three doses, the induced immune responses remained high for 70 days. While antibodies induced after immunization with N-terminal and central regions showed similar affinities to the target antigens, affinities of IgG against PmMSP1_19_ were higher. All proteins induced similar antibody subclass patterns (predominantly IgG1, IgG2a, and IgG2b), characterizing a mixed Th1/Th2 response. Further, autologous stimulation of splenocytes from immunized mice led to the secretion of IL2 and IL4, independently of the antigen used. Importantly, IgG from *P*. *malariae*-exposed individuals reacted against PmMSP1 recombinant proteins with a high specificity. On the other hand, sera from *P*. *vivax* or *P*. *falciparum*-infected individuals did not react at all against recombinant PmMSP1 proteins.

**Conclusion:**

Recombinant PmMSP1 proteins are very useful diagnostic markers of *P*. *malariae* in epidemiological studies or in the differential diagnosis of malaria caused by this species. Immunization with recombinant PmMSP1 proteins resulted in a significant humoral immune response, which may turn them potential component candidates for a vaccine against *P*. *malariae*.

## Introduction

*Plasmodium malariae* was the first malaria parasite observed in 1881 by Charles Laveran, Nobel Prize winner for discovering the cause of malaria [1]. *P*. *malariae* is a cosmopolitan parasite that does not develop below 15°C and is described in tropical and subtropical regions of sub-Saharan Africa, South America, much of Southeast Asia, Indonesia, and in many islands of the western Pacific. In general, the distribution coincides with that of *P*. *falciparum*. In South America, *P*. *malariae* is associated with zoonotic infections, and is easily confounded with *P*. *brasilianum*, a parasite that infects twelve different genera of New World primates [1]. These and humans have high levels of seropositivity for *P*. *malariae* and *P*. *brasilianum* antigens in endemic areas [2].

Parasitemias in *P*. *malariae* infections are usually low compared to those in patients infected with *P*. *falciparum* or *P*. *vivax* perhaps due to its longer developmental cycle (72 h for *P*. *malariae* versus 48 h for *P*. *vivax* and *P*. *falciparum*), lower number of merozoites produced per erythrocyte cycle, and its preference for developing in older erythrocytes [2]. The combination of these factors may be a trigger for the earlier development of an immune response by the host [2]. *P*. *malariae* may persist asymptomatically in the infected individual for long periods [3], but it may also result in sequelae after decades without being perceived by the carrier [4]. Unlike *P*. *vivax* and *P*. *ovale* infections, there is no evidence of latent liver stage forms (hypnozoites) [2].

Previous studies point to *P*. *malariae* as one of the oldest parasites that cause malaria. The adaptive success is characteristic of the slow biological cycle that favors its survival for long periods in the host organism without being noticed [1]. Although rarely associated with severe malaria, there are cases of nephrotic syndrome related to *P*. *malariae* (rev. in [2]) and cases of death in immunocompromised patients [5,6]. Also, treatment failures [7,8] and acute renal injury [9] are attributable to infection caused by *P*. *malariae*. It is important to note that this silent survival for long periods in the organism makes this parasite the most important in transfusion malaria [2,5,10,11,12]. Additionally, is difficult to detect and, consequently, to control. In this sense, an effective vaccine would be a valuable addition to the tools available for malaria control.

Merozoite surface protein 1 (MSP1) is one of the major candidates for an antimalarial vaccine because it is expressed abundantly on the merozoite surface and capable of activating the host protective immunity [13]. Studies using MSP1 in different endemic regions have shown that this protein possesses highly immunogenic fragments that generate an immune response, which frequently associates to protection in natural infections (rev. in [14]). While many studies that deal with MSP1 from *P*. *vivax* and *P*. *falciparum* have demonstrated a strong immune response in mice and monkeys [15,16], *P*. *malariae* MSP1 has not been examined so far.

In order to investigate the immunogenicity of the *P*. *malariae* MSP1 polypeptide, we evaluated the humoral and cellular immune responses of BALB/c mice immunized with five recombinant proteins, representing different portions of the PmMSP1 protein.

## Materials and methods

### PmMSP-1 recombinant proteins

**Fig 1:**
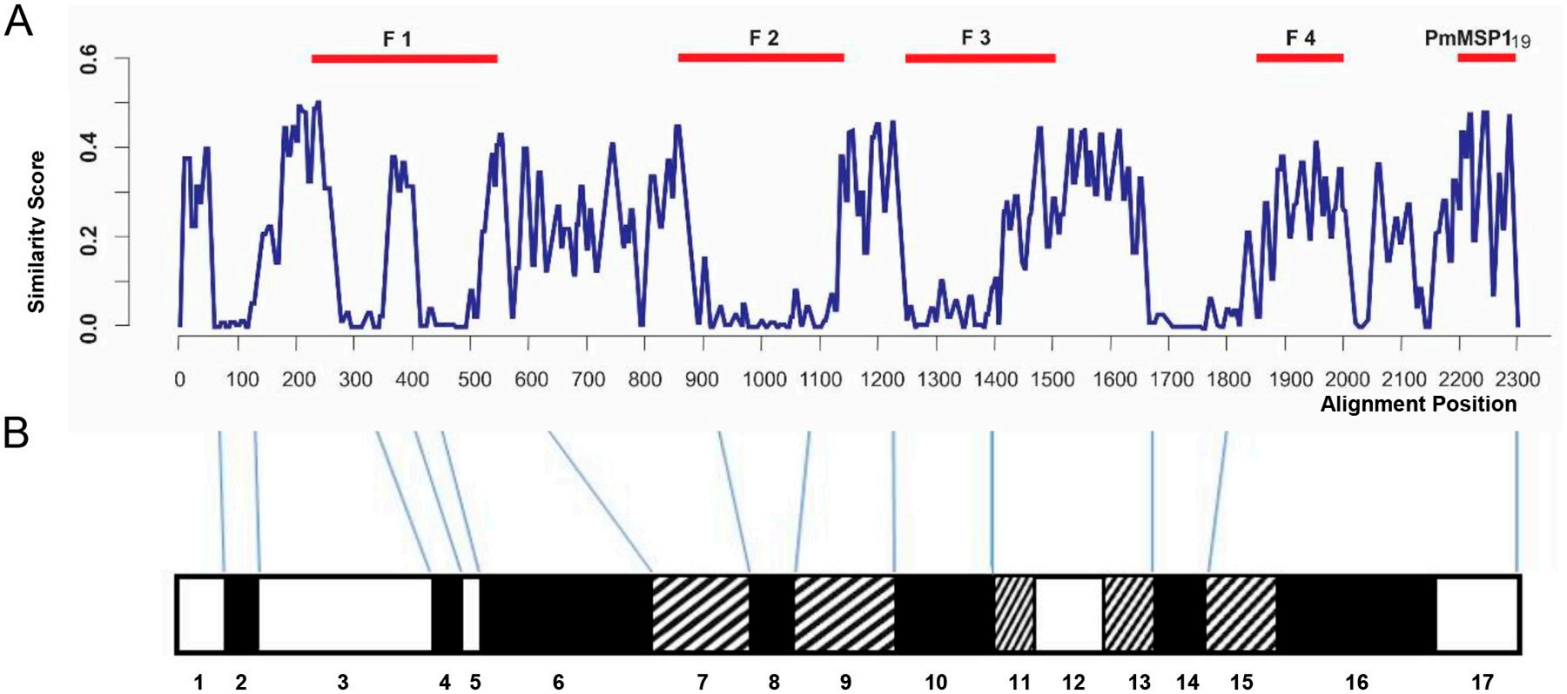
Comparison of MSP1 amino acid sequences of 16 species of *Plasmodium*. The location, size, and variability of each fragment of PmMSP1 (F1, F2, F3, F4 and PmMSP1_19_) used in this work are represented (modified from [18]) **(A)**. Positioning of A in relation to the PfMSP1 blocks, where sequences are divided into conserved blocks (white boxes), semi-conserved blocks (hatched boxes), and variable blocks (black boxes) (modified from [19] **(B)**.

The PmMSP1 fragments were obtained from sample I11, isolated in 2002 from an infected patient in Iporanga, a municipality located in Atlantic Forest Region of the State of São Paulo, Brazil [17]. Five fragments were selected by representing conserved and polymorphic regions of the N-terminal, C terminal and the central portion of the protein (Fig 1A) (modified from [18]). The positioning of these fragments in relation to the PfMSP1 blocks, where sequences are divided into conserved blocks (white boxes), semi-conserved blocks (hatched boxes), and variable blocks (black boxes) is shown in Fig 1B (modified from [19]). Primer sequences and amplification conditions used were previously described [18]. The purified fragments were ligated into the pGEM-T Easy vector (Promega) according to the manufacturer’s instructions and subsequently subcloned into the pGEX-3Y vector (pGEX-3X altered by fill-in modification of the *Bam*HI site). Recombinant plasmids were checked for their integrity by semiautomatic sequencing using the Big Dye Terminator v3.0 Cycle Sequencing Kit in an ABI Genetic Analyzer 3550 (ABI, USA). The sequences were deposited in GenBank (F1, #KR072279; F2, #KY189271; F3, #KY189272; F4, #KY189273; PmMSP1_19_, #KY189274). The recombinant PvMSP1_19_ and PfMSP1_19_ proteins corresponding to the amino acid sequence of the 19-kDa fragment of MSP1 of the isolates Belém and S-20, respectively, were used as controls [20,21]. Then, the expression and purification of all recombinant proteins were performed as previously described [21].

### Immunization of mice with the recombinant proteins

**Fig 2:**
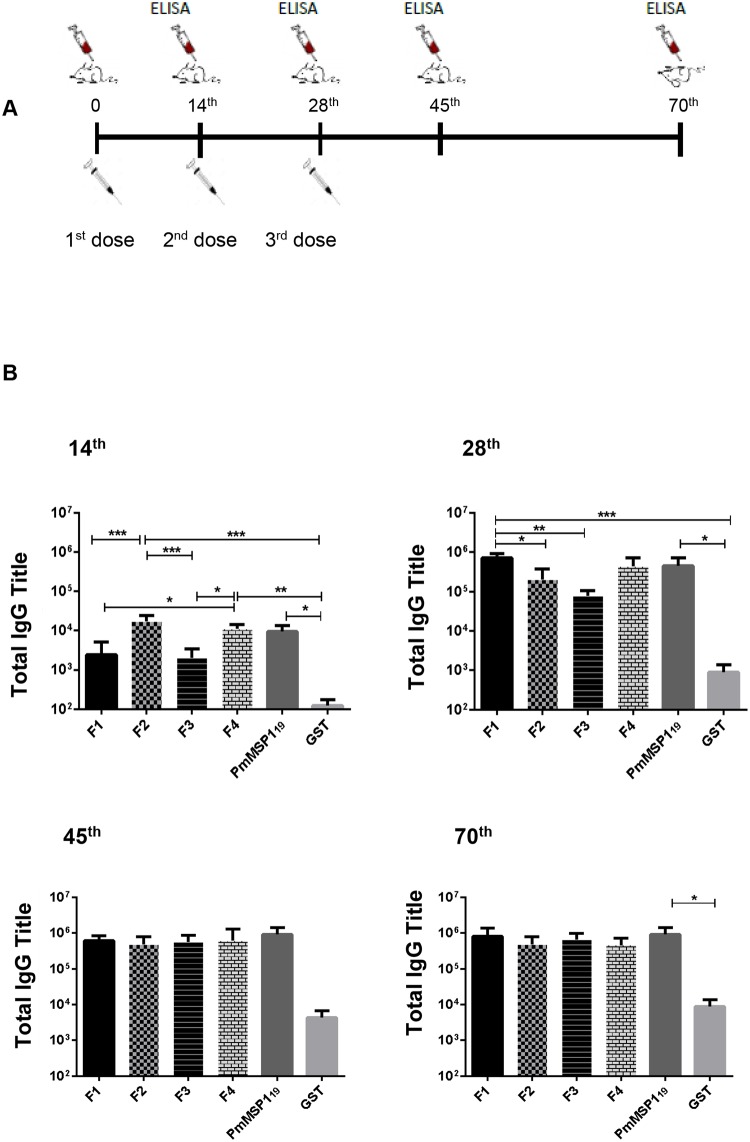
Schematic representation of collections of blood, immunizations, and tests performed. The blood samples were collected before each immunization. On the 70th day, the animals were sacrificed and the spleens removed for the CBA and lymphoproliferation assays **(A)**. Immune response of IgG antibodies in mice immunized with recombinant proteins produced in *E*. *coli*. Groups of four BALB/c mice were immunized intraperitoneally with 25 μg of the indicated recombinant proteins emulsified in Freund adjuvant plus MPLA **(B)**. Antibody titers were detected by ELISA in the 14^th^, 28^th^, 45^th^ and 70^th^ day after the first immunization. The results are expressed by geometric mean ± standard deviation. * (p <0.05), ** (p <0.001) and *** (p <0.0001).

For all experiments, 6 groups (F1, F2, F3, F4, PmMSP1_19_ and GST as control) of four female 6–8 weeks old BALB/c mice were used. The immunization was performed via the intraperitoneal (i.p.) route with 25 μg of recombinant protein in the presence of Freund’s incomplete adjuvant (Sigma) and 500 ng monophosphoryl lipid A adjuvant (MPLA) (Avanti)/dose. Three doses were administered at 15-day intervals. Blood samples were collected by a puncture of the submandibular artery of the immunized animals. Blood samples were collected before the first immunization and at the 14th, 28th, 45th and 70th day after the first immunization (Fig 2A). The blood was incubated at 37°C for 1 h, then at 4°C for 1 h and centrifuged at 12,000 rpm for 10 minutes at 4°C. Resulting serum was separated in new tubes and stored at -20°C.

### ELISA for detection of mouse antibodies against PmMSP1 recombinant proteins

Total IgG antibodies against F1, F2, F3, F4, PmMSP1_19_ and GST in mice sera were detected by ELISA 14, 28, 45 and 70 days after the first immunization, as described previously [22]. Recombinant antigens were added to the plates at 250 ng/well and the secondary antibody (peroxidase-coupled goat anti-mouse IgG [KPL]) was used in a 1:5000 dilution. Each serum was analyzed in serial dilutions starting from 1:200. The titers were determined as the highest dilutions presenting optical density (OD) at 450 nm (OD_450_) values higher than 0.1. These values represent binding of IgG to the recombinant protein after subtraction of binding of the same serum to GST alone. The results are presented as the geometric mean ± standard deviation of four animals per group.

Detection of IgG subclass responses was performed as described above, except that the secondary antibodies were specific for mouse IgG1, IgG2a, IgG2b and IgG3 (all obtained from Bethyl Laboratories) diluted 1:5,000. The OD values of the sera against the fusion proteins were corrected by subtracting the OD values from the GST-coated wells.

The affinities of the antibodies were assessed by a thiocyanate elution-based ELISA [23]. The procedure was similar to that described for the standard ELISA with the inclusion of an extra step. After washing of plates following incubation of the pooled serum dilutions of the sample after the 3rd immunization (1:25,600), ammonium thiocyanate, diluted in PBS, was added to the wells in duplicate, in concentrations ranging from 0 to 8 M. The plates were allowed to stand for 15 min at room temperature before they were washed and incubated with the secondary antibody solution. Afterwards, the concentration of ammonium thiocyanate required to dissociate 50% of the bound antibody was determined. The percentage of binding was calculated as follows: OD_492_ in the presence of ammonium thiocyanate x100 / OD_492nm_ in the absence of ammonium thiocyanate.

### Determination of cytokine concentration in spleen cell supernatants

Cytokine levels secreted into the splenocyte culture supernatant stimulated for 72 h with the recombinant proteins (20 μg/mL) or ConA (2.5 μg /mL), were determined using a mouse Th1/Th2/Th17 cytometric bead array kit (CBA; BD Biosciences) on a LSR Fortessa BD flow cytometer in accordance with manufacturer’s instructions and data analysis was performed by FCAP Array v.3 software.

### Human serum samples

Serum samples from 15 healthy individuals and 21 individuals with unrelated diseases (3 schistosomiasis, 5 visceral leishmaniasis, 5 Chagas disease, 2 toxoplasmosis, 2 rheumatoid arthritis, 4 lupus) were obtained from the Biorepository of Samples, Laboratory of Protozoology, Tropical Medicine Institute, University of São Paulo. Serum samples from 16 individuals with malaria were obtained from the Biorepository of Samples of the Department of Parasitology, Biomedical Sciences Institute, University of São Paulo. Ethical clearance for the extension of the use of these sera was obtained from the Ethics Commission (Biomedical Sciences Institute, USP, CEPSH 20/2015, n° 1.003.485).

### Immunoblotting assay for PmMSP1 detection

For this analysis, the recombinant proteins were separated by SDS-PAGE (12%) diluted (vol/vol) in SDS-sample buffer (60 mM Tris-HCl [pH 6.8], 5% 2-mercaptoethanol, 10% glycerol, and 0.01% bromophenol blue), and boiled for 5 min at 100°C. Samples were loaded on a 12% polyacrylamide minigel (Mini-Protean II; Bio-Rad). Antigens were separated by electrophoresis, transferred to nitrocellulose membranes (Hybond-C, GE Healthcare), and blocked with PBS containing 5% non-fat milk for 1 h at room temperature. Membranes were incubated with a serum pool of *P*. *malariae* patients (1:500) diluted in PBS with 1% milk. Bound antibodies were detected with horseradish peroxidase-labeled anti-human IgG (Sigma Co.). The color of detected bands was developed after reaction using a chemiluminescence detection assay (Kit Western Pico Super Signal, Pierce/Thermo Scientific).

### Statistical analysis

Statistical analysis was performed using the GraphPad Prism program version 5.0 for Windows (GraphPad Software, USA). For the comparison of antibody titers of more than two groups at the same sampling time ANOVA analysis was used, and *a posteriori* multiple comparisons between the means of the groups were performed with Tukey’s Test. To determine the differences between the sampling times ANOVA double-ranking was used, followed by a Bonferroni test. The cutoffs were established by using the mean plus three standard deviations of signal measured with negative sera.

### Ethics statement

The mice used in this study were provided by the Department of Parasitology, Institute of Biomedical Sciences. All the procedures were submitted to the Ethics Committee of the Institute of Medicine Tropical and were approved under the registration number of the Committee on Ethics in the Use of Animals (CEUA) (CPE-IMT / 000304A). All animals immunized were females aged from 4 to 6 weeks and were maintained in a 12 h light/12 h dark cycle with free access to water and standard chow under appropriate environmental and hygienic conditions. Serum was harvested by submandibular vein bleeding. After trials, mice were euthanized using CO_2_ asphyxiation as recommended [24]. Ethical clearance for the extension of use of sera from the plasma/sera collection at the Department of Parasitology was obtained before the experimental work (CEPSH protocol 20/2015 and extension No. 1.003.485).

## Results

### Immunogenicity of five different PmMSP1 recombinant proteins in mice

To characterize the immune responses generated by immunization with PmMSP1, immunogenicity studies in mice were conducted. Groups of 4 BALB/c mice were immunized with F1, F2, F3, F4, PmMSP1_19_ and with GST alone as a control, and the resulting antibody responses were measured by ELISA using recombinant proteins. Results in Fig 2B (a-d) show that all the recombinant proteins were immunogenic.

The time course of the immunizations and antibody titers showed that the responses to F1, F2, F3, F4, PmMSP1_19_ were boosted after the second immunization while specific antibody titers were similar (1: 809,000) after the third immunization and were maintained until the end of the experiment (day 70) (S1 Fig) (for the Prism files that generate the figures in the paper see Raw Data in S1 File).

**Fig 3:**
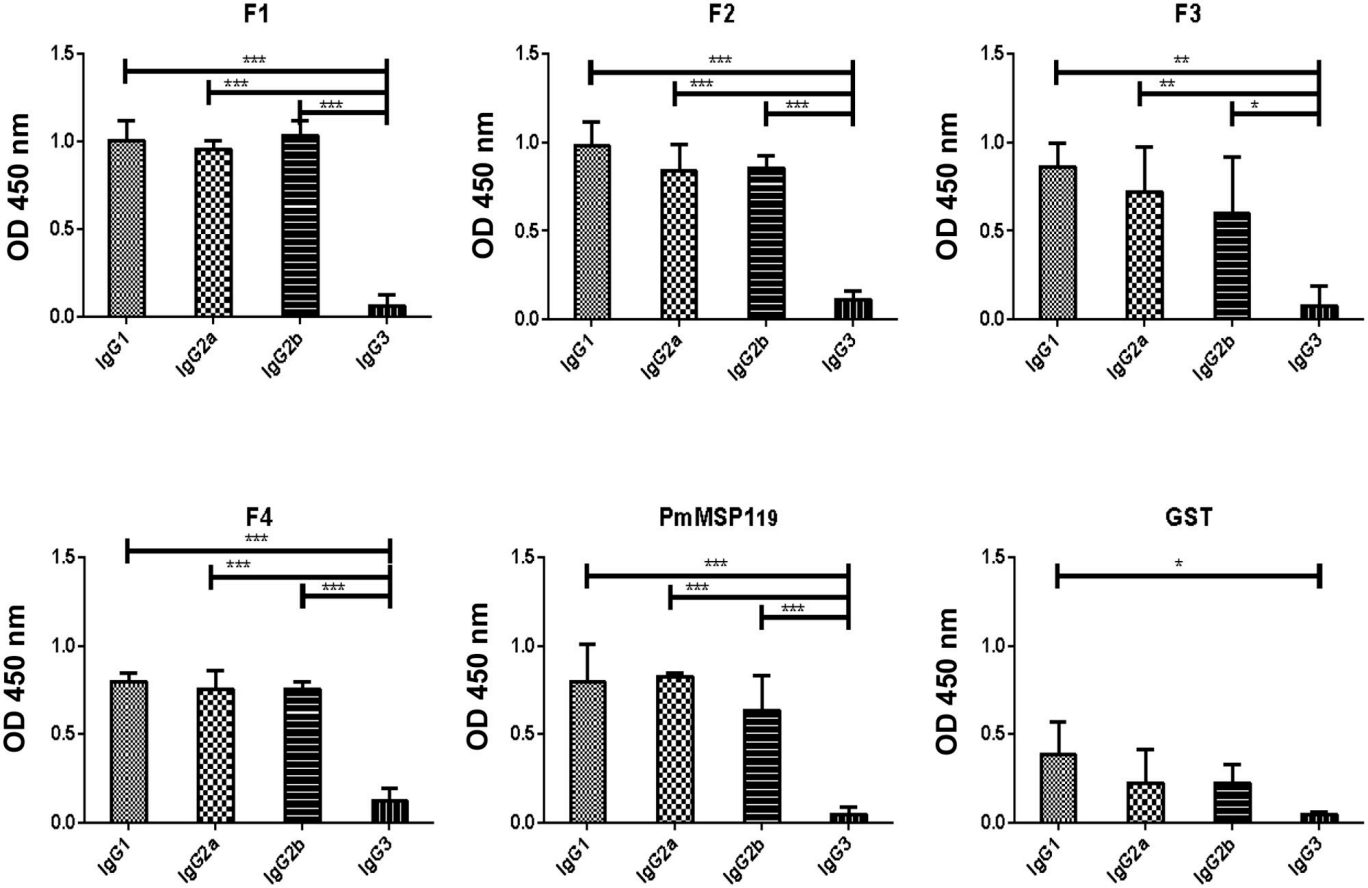
Analysis of specific IgG subclasses. F1, F2, F3, F4, PmMSP1_19_ and GST, in the different groups of mice immunized with the protein fragments on the 45th day after immunization at a dilution of 1: 1200. Subclass levels IgG1, IgG2a, IgG2b and IgG3 in the sera of the animals were measured by ELISA. The results are expressed by geometric mean ± deviation pattern. * (p <0.05), ** (p <0.001) and *** (p <0.0001).

The isotype distribution of IgG responses to F1, F2, F3, F4 and PmMSP1_19_ showed that all recombinant proteins elicited similar patterns of IgG subclasses. Antibodies of all isotypes were detected with the levels of IgG1, IgG2a and IgG2b higher when compared to IgG3 (Fig 3). However, no significant differences were observed in the mean OD values between IgG1, IgG2a or IgG2b. The data presented here correspond to pooled sera collected at day 45.

**Fig 4:**
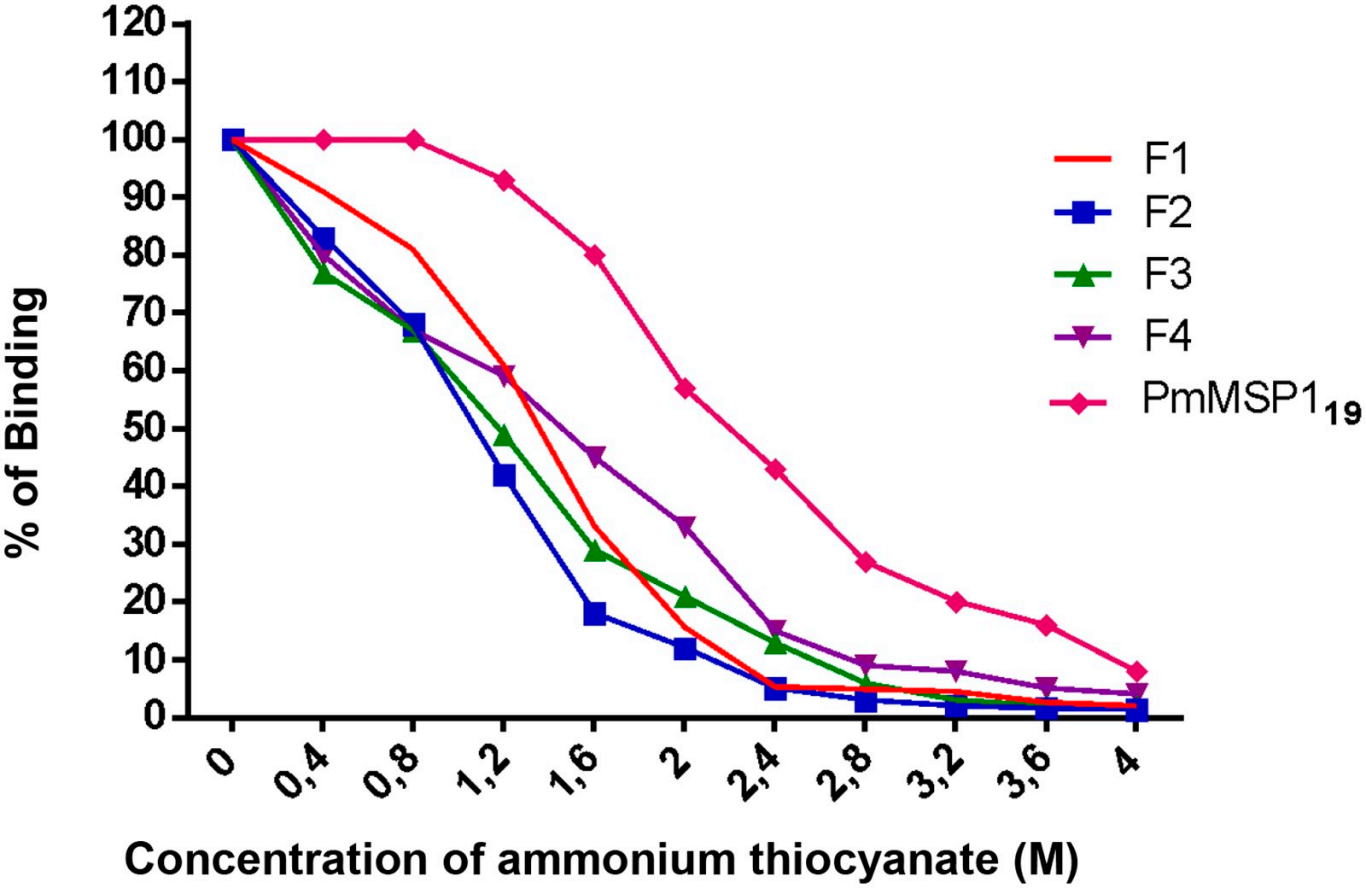
Determination of the affinity by ELISA of specific anti-F1, anti-F2, anti-F3, anti-F4, anti-PmMSP1_19_ IgG antibodies based on the elution method with ammonium thiocyanate. The pooled sera of 4 BALB/c mice immunized intraperitoneally each of the recombinant proteins were evaluated in the presence of increasing concentrations (M) of ammonium thiocyanate. The results are expressed as the average of duplicates.

The sera obtained were used to determine their affinity for corresponding recombinant proteins. These antibodies induced after immunization with the recombinant proteins F1, F2, F3 and F4 showed similar affinities since the concentrations of ammonium thiocyanate required for dissociating 50% of the antibodies were similar (1.279 M, 1.087 M, 1.216 M and 1.391 M, respectively). Interestingly, the observed affinity was higher (2.096 M) for PmMSP1_19_ (Fig 4).

### Lymphoproliferation assays

**Fig 5:**
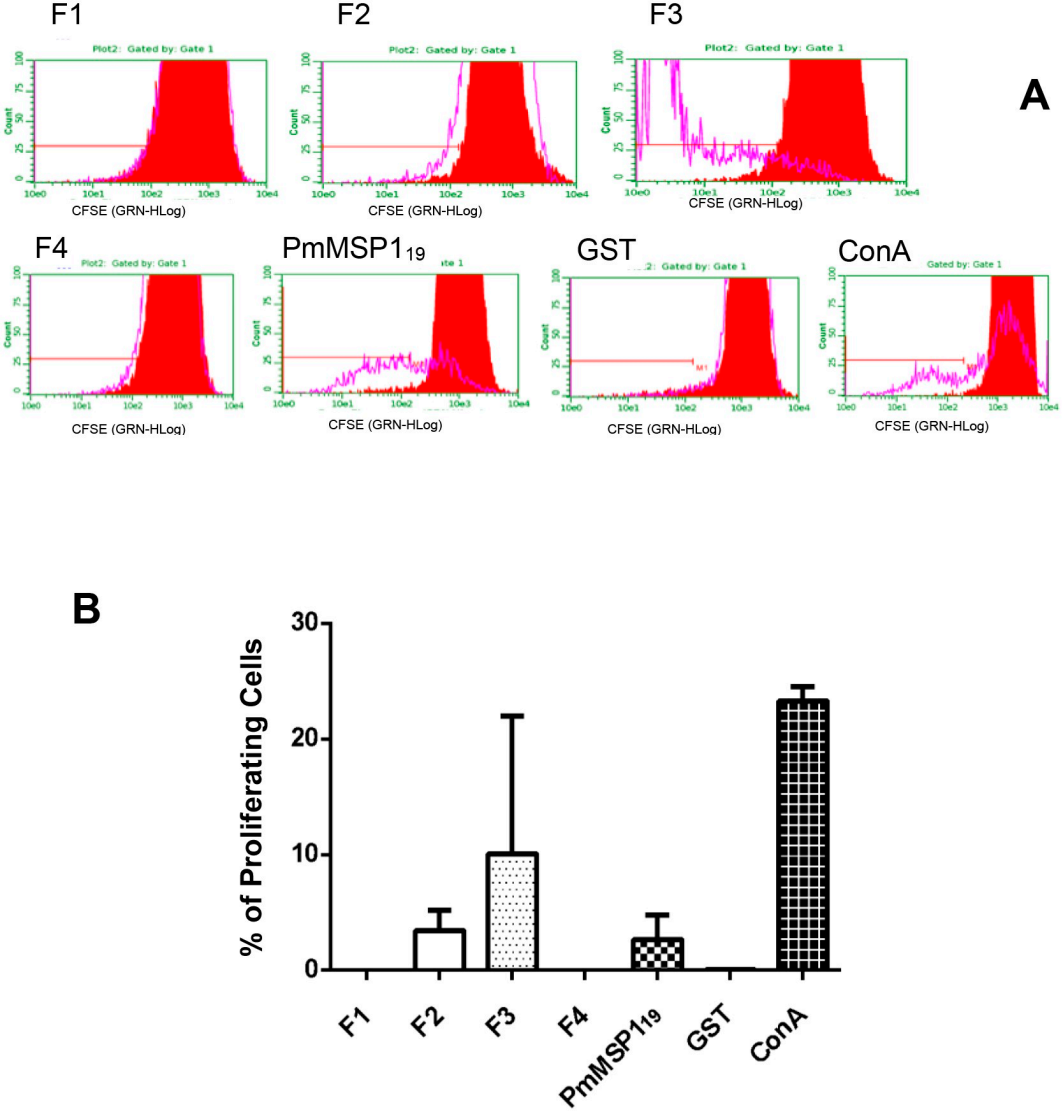
GST-fused F2, F3, and PmMSP1_19_ lead to lymphocyte proliferation in spleen cells from immunized mice. Splenocytes of immunized BALB/c mice were stimulated with 20 μg/ml of each recombinant protein used in the immunizations. The cells were stimulated for 72 hours and the fluorescence emitted by CFSE was monitored using a flow cytometer. Representative result (**A)** of the F1, F2, F3, F4, PmMSP1_19_, GST and concanavalin A. Data were measured in triplicate and transformed in percentage of proliferating cells **(B)**.

The proliferation of lymphocytes stimulated with each antigen was determined by flow cytometry, using CFSE as a proliferation indicator. Concanavalin A stimulation was used to demonstrate the viability of cells used in this experiment (positive control). In Fig 5A, representative results of the F1, F2, F3, F4, PmMSP1_19_, GST and concanavalin A stimulation are shown. In Fig 5B, data were measured in triplicate and transformed into percentage of proliferating cells. As expected, a high percentage of proliferation was obtained with concanavalin A. Cells stimulated with F2, F3 and PmMSP1_19_ showed a progressive decrease in fluorescence after 72 h, indicating proliferation. Almost no proliferation was observed for GST-, F1- and F4-peptide stimulation (Fig 5B).

### Stimulated lymphocytes secrete IL4

**Fig 6:**
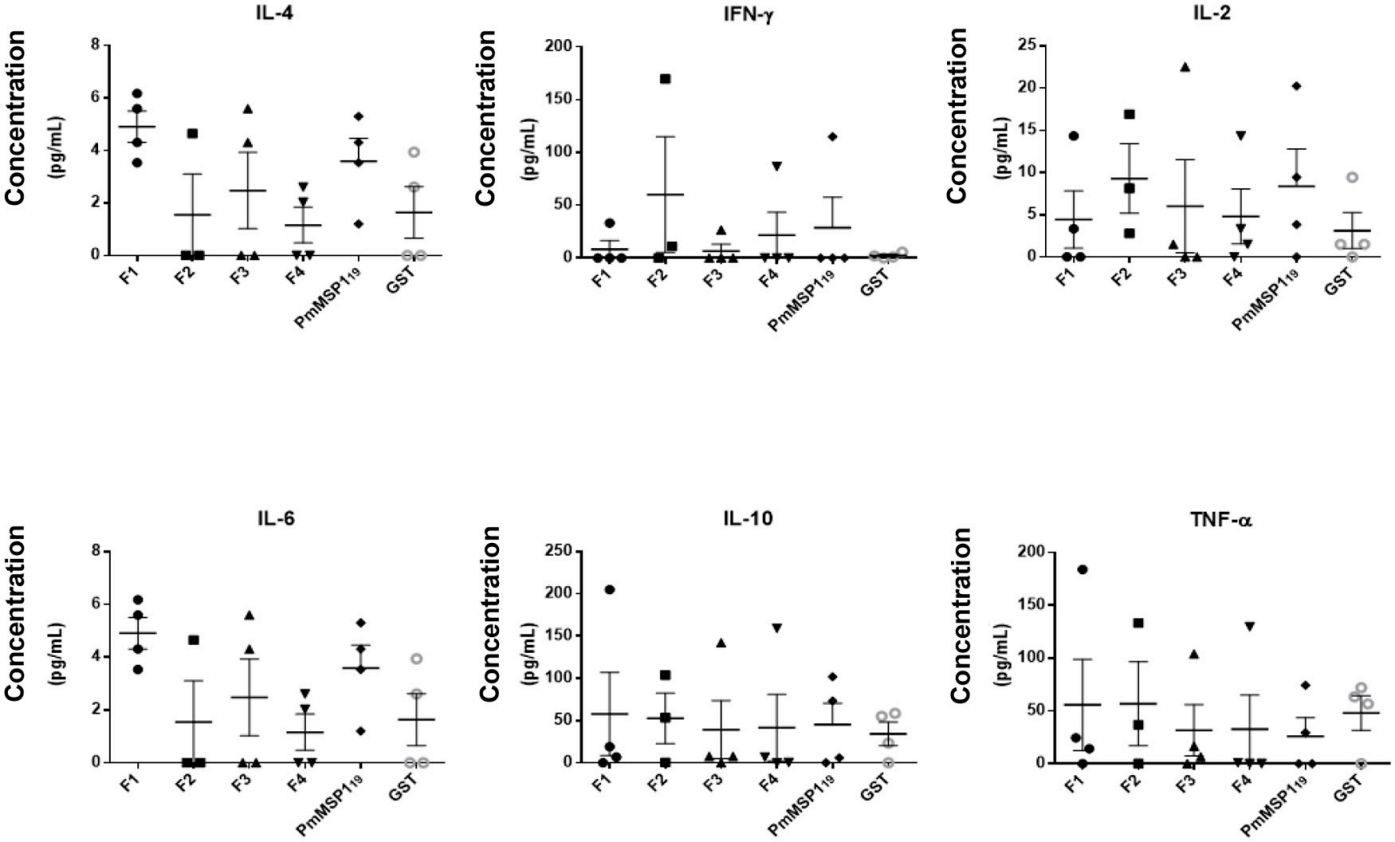
Effect of recombinant proteins on the Th1/Th2 ratio. Levels of cytokines IL-4, IFN-γ, IL-2, IL-6, IL-10 and TNF-α, in the supernatants of splenocytes, stimulated for 72 hours in the different groups of mice immunized.

After antigenic stimulation, levels of cytokines secreted in the culture supernatants of the splenocytes were determined by CBA (Fig 6). The levels of Th1/Th2 cytokines detected did not show significant differences when compared between the groups, but we observed that the level of IL-4 in the groups F1 and PmMSP1_19_ was higher than in the other groups (Fig 6). Due to the low levels of cytokines IFN-γ, IL-2, IL-6, IL-10 and TNF-α (Fig 6) found in most animals, differences in these results are not statistically significant.

### Patient sera from natural infections recognize PmMSP1 recombinant proteins

**Fig 7:**
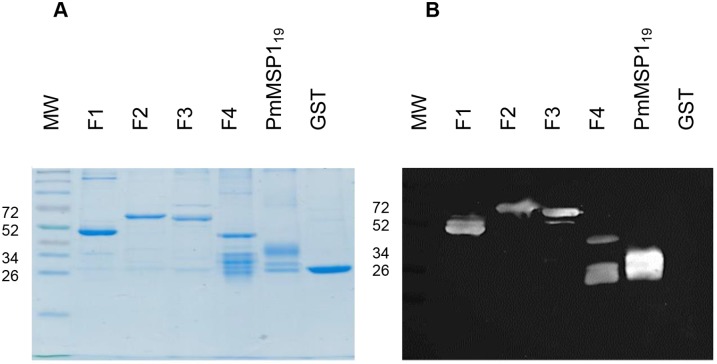
SDS-PAGE electrophoresis and Western blot. Recombinant proteins were subjected to 12% SDS-PAGE electrophoresis and stained with Coomassie Blue **(A)**. MW: Molecular weight standard (kDa), F1, F2, F3, F4, PmMSP1_19_ and GST; Western blot showing the recognition of proteins by a pool of sera from *P*. *malariae* patients **(B)**. MW: Molecular weight standard (Thermo/Fermentas broad range prestained marker, weights in kDa), F1, F2, F3, F4, PmMSP1_19_ and GST.

The identity and specificity of the recombinant proteins were analyzed by Western blot using a pool of sera from patients positive for *P*. *malariae* confirmed by PCR (Fig 7). The results showed a migration pattern specific and similar in the SDS-PAGE (Fig 7A). Recombinant proteins of interest, F1, F2, F3, F4, and PmMSP1_19_, reacted with human serum samples of patients with malaria caused by *P*. *malariae* (Fig 7B). GST alone was not recognized by sera from *P*. *malariae* infected patients (Fig 7B).

### PmMSP1 proteins are not cross-recognized by sera from *P*. *vivax* or *P*. *falciparum* infected patients, and vice versa

**Fig 8:**
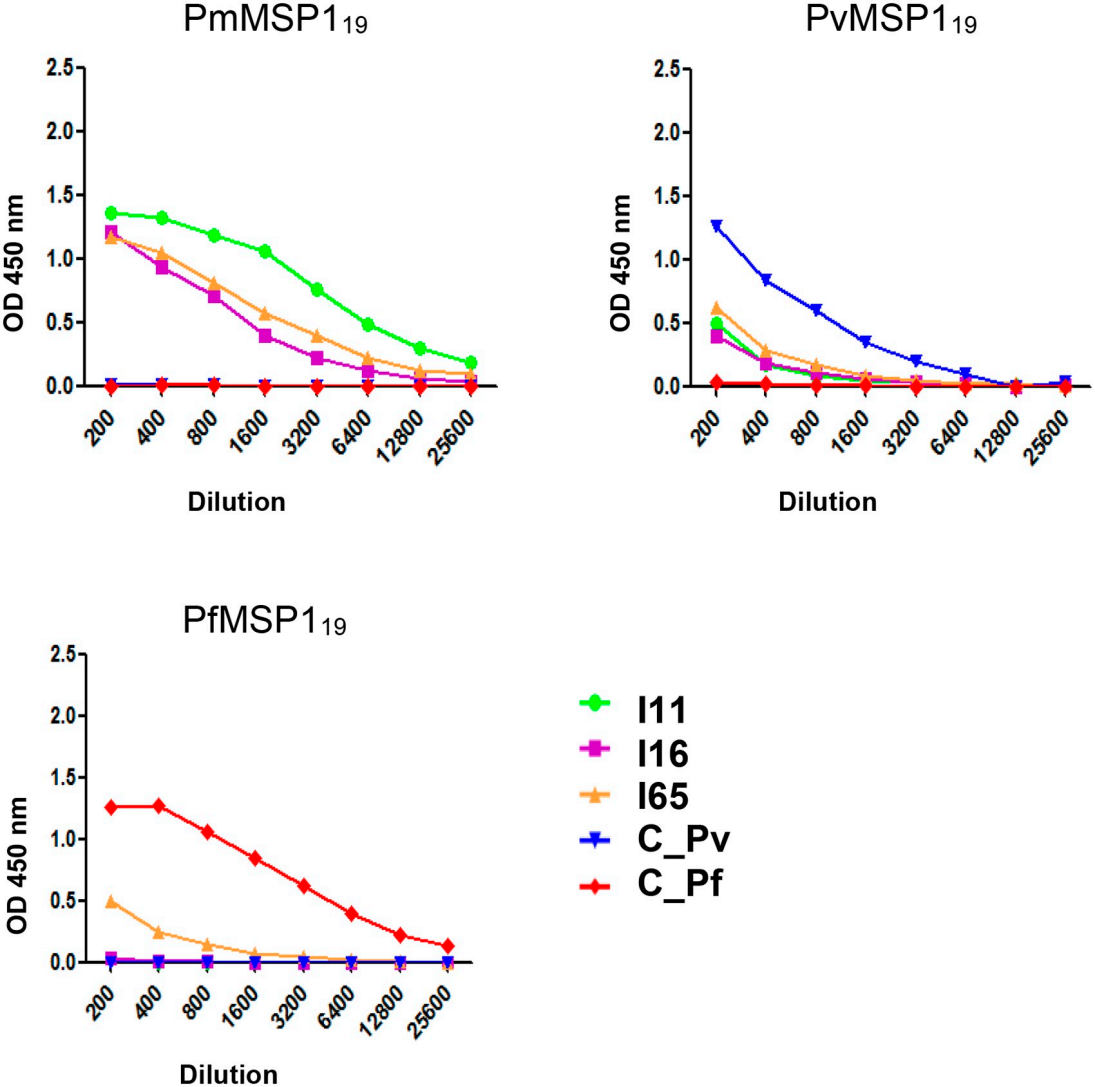
Reactivity by ELISA of recombinant proteins. PmMSP1_19_, PvMSP1_19_ and PfMSP1_19_ proteins, with sera from *P*. *malariae* (I11, I16 and I65), *P*. *vivax* (C_Pv) or *P*. *falciparum* (C_Pf) positive patients.

The reactivity of MSP1_19_ proteins of *P*. *malariae* (PmMSP1_19_), *P*. *vivax* (PvMSP1_19_) and *P*. *falciparum* (PfMSP1_19_) was evaluated by ELISA with five patient sera. As shown in Fig 8, when the ELISA plate was sensitized with PmMSP1_19_, only the sera from patients I11, I16, I65 (infected with *P*. *malariae*) recognized the antigen (Fig 8, PmMSP1_19_). When the ELISA plate was sensitized with PvMSP1_19_ (Fig 8, PvMSP1_19_) or PfMSP1_19_ (Fig 8, PfMSP1_19_), only the sera from patients with *P*. *vivax* (C_Pv) or *P*. *falciparum* infection (C_Pf) reacted with their corresponding antigens. Some recognition by *P*. *malariae* patient sera was also recorded, possibly due to previous, not reported, contact with *P*. *vivax* and *P*. *falciparum*.

**Fig 9:**
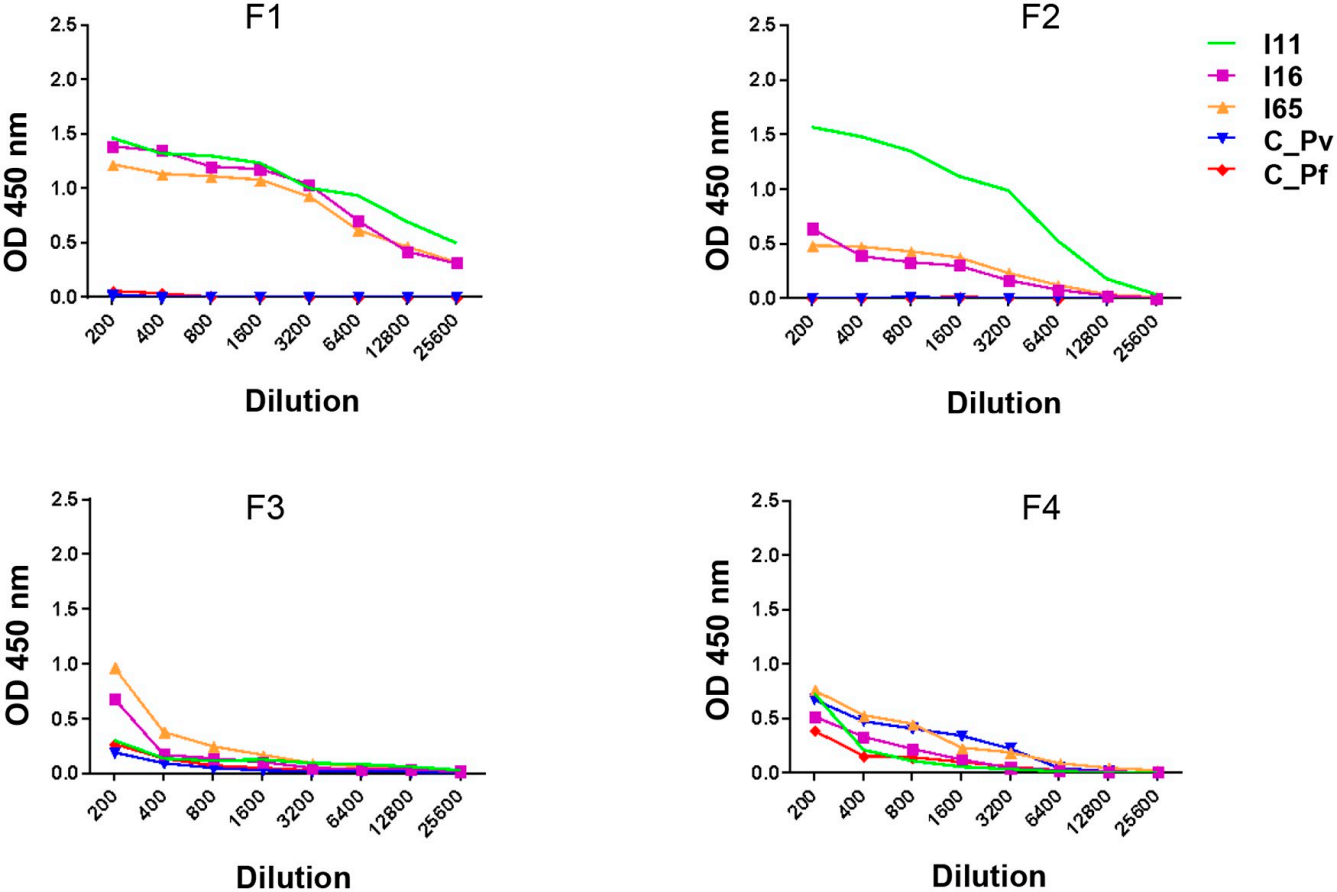
Reactivity by ELISA. F1, F2, F3 and F4 recombinant proteins in sera from *P*. *malariae* (I11, I16 and I65), *P*. *vivax* (C_Pv) or *P*. *falciparum* (C_Pf) positive patients.

We also evaluated the reactivity of the recombinant proteins F1, F2, F3 and F4 with those five sera belonging to patients confirmed with *P*. *malariae*, *P*. *vivax* and *P*. *falciparum* by ELISA (Fig 9). Although our primary goal with this small study was to show the natural antibody response acquired that is induced against the different MSP1 fragments, we found a stronger recognition of F1 (Fig 9, F1) than that obtained for the F2, F3 and F4 recombinant proteins (Fig 9, F2, F3 and F4). It was also possible to observe a higher recognition of F2 by the homologous serum than by the heterologous sera (Fig 9, F2).

**Fig 10:**
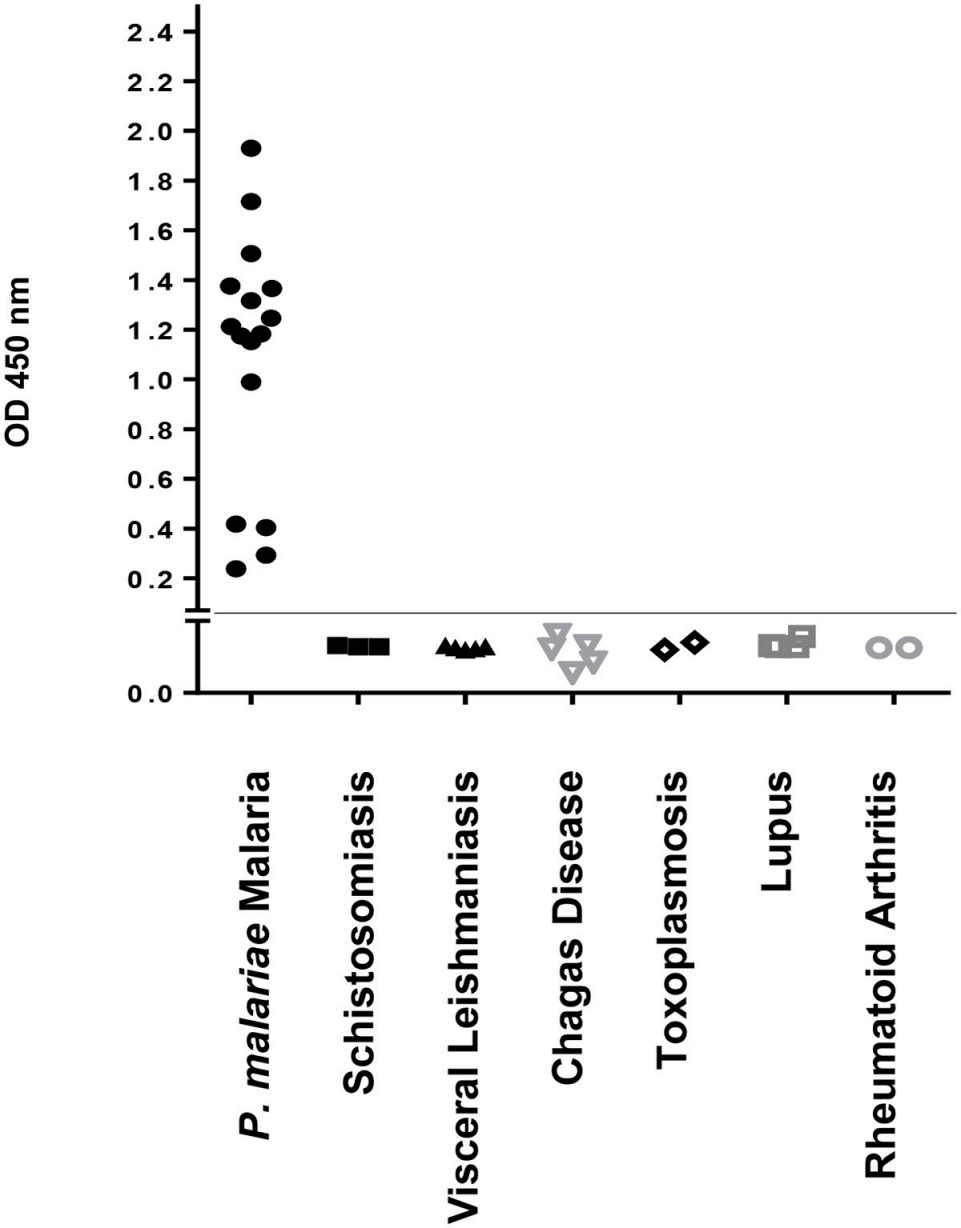
Reactivity by ELISA of PmMSP1_19_ by IgG antibodies in samples from sera from 16 individuals diagnosed with *P*. *malariae* or unrelated diseases. The line represents the *cutoff* value (OD 0.07) established at three standard deviations above the mean OD_450_ of sera from 15 individuals, never exposed to malaria, from the city of São Paulo.

MSP1_19_ from *P*. *malariae* was recognized by sera from malaria patients caused by *P*. *malariae* (100% of responders, n = 16) (Fig 10). The corresponding IgGs were species-specific and no reaction was detected with 21 sera from individuals with unrelated diseases (specificity of 100%) (Fig 10). Cut-off points (OD 0.07) were set at three standard deviations above the mean OD_450_ of sera from 15 individuals, never exposed to malarial infection, from the city of São Paulo. The reactivity obtained with the sera of patients from other diseases was similar to that obtained with sera from healthy individuals. This proves that the herein produced PmMSP1 antigens are very useful when a specific humoral response against *P*. *malariae* is required.

## Discussion

The development of effective methods for malaria diagnosis and control is still on the malaria research agenda. There is currently one vaccine (RTS,S/AS01) being applied in some African countries (https://www.who.int/news-room/detail/23-04-2019-malaria-vaccine-pilot-launched-in-malawi). Of the 25 malaria vaccine projects under development in the world, four are in stage IIb or III [25,26]. All but one candidate are targeted exclusively against *P*. *falciparum*, which is probably insufficient in areas where a significant proportion of patients suffers from *P*. *vivax* or mixed infections. Furthermore, there are three other species that infect humans that have to be considered: *P*. *malariae*, *P*. *ovale*, and *P*. *knowlesi*.

*P*. *malariae* has a much lower incidence than *P*. *falciparum* and *P*. *vivax*, but unlike *P*. *ovale* and *P*. *knowlesi*, it occurs in all endemic areas of malaria, coexisting with other species, and should, therefore, be considered in the control strategies. In fact, there are no reports to date that study the immune response to *P*. *malariae* vaccine candidate antigens. Although the *P*. *malariae* merozoite surface protein 1 was isolated and characterized nine years ago [27], this is the first report showing the immunogenicity of five recombinant forms of this protein in BALB/c mice and their recognition by sera from individuals living in malaria-endemic regions of Brazil. Different regions of PmMSP1, covering many parts of the protein, encompassing polymorphic and conserved regions, were fused to glutathione S-transferase (GST). Fusion of GST to the N-terminus of proteins facilitates their purification and often helps to maintain fused polypeptide soluble compared to a 6xHistidine-fusion. The expression system in *E*. *coli* was chosen because of its simplicity, cost-effectiveness and high efficiency in the expression of non-glycosylated proteins [28,29]. The recombinant proteins produced were used to immunize BALB/c mice using monophosphoryl lipid A adjuvant (MPLA) and Freund’s incomplete adjuvant. The specificity and subtyping of the antibodies and cellular immune responses were evaluated by ELISA and cytometry, respectively, using the recombinant proteins as antigens.

### Immunogenicity of PmMSP1 recombinant proteins in mice

Regarding the response of antibodies against blood stage antigens, known for their importance in protecting against malaria [30], our results demonstrate that the recombinant proteins were immunogenic in mice. The titers of IgG antibodies elicited by F1, F2, F4, and PmMSP1_19_ were significantly higher than the antibody titers found in the control group (GST). After the second dose, the most immunogenic proteins were the F1 (N-terminal) and PmMSP1_19_ (C-terminal) regions. These regions were also found to be the most immunogenic in other species of *Plasmodium*, such as *P*. *vivax*, where, using recombinant plasmids for the immunization of mice, the most antigenic plasmids were those that encoded the N-terminal and C-terminal regions [20].

Antibodies to MSP1 may persist for up to 30 years in some patients with malaria [31]. Although several studies have shown that a high level of total IgG against total antigens of the *P*. *falciparum* blood stage is a bad predictor of protection [32], it is believed that different functions are performed by each subclass of IgG in the acquisition of immunity to the malaria parasites [33]. The development of vaccines against malaria in the blood stage focuses on the proteins expressed on the surface of the parasite or infected erythrocytes, which are accessible for antibodies. Since both the cellular and the humoral response are important against malaria parasites (rev. in [34]), parameters of both responses were monitored after immunization. An analysis of generated antibodies demonstrated that the four subclasses of IgG were detected on the 45th day after the first immunization of the mice immunized in all groups. The five recombinant proteins induced similar antibody patterns, being IgG1, IgG2a and IgG2b the most predominant. Previous studies have shown that immune responses induced by PvMSP1_19_ antigens [35,36] or PfMSP1_19_ [37,38,39] in mice confer high levels of IgG1 subclasses, followed by IgG2a and IgG2b. Others authors also found similar results with recombinant proteins derived from PvMSP9 [40]. It is important to note that IgG2a antibodies from mice are considered the most effective in complement activation and in the activation of antibody-dependent cellular cytotoxic mechanisms, in addition to modulating the parasitemia of *P*. *yoelii* [41,42]. In our study, the recombinant proteins in emulsion with the adjuvant induced a mixed Th1/Th2 response also found in other studies [43]. Different studies have also shown that, in *P*. *yoelii*, protection occurs in the presence of elevated levels of IgG1, IgG2a and IgG2b, with a mixed Th1/Th2 immune response [44,45,46,47].

Cytokines play a critical role in determining the subclasses of IgG. IL-4 is secreted by Th2 cells and is associated with an IgG1 response, an indicator of a predominantly antibody-mediated response. As demonstrated by our results, the spleen cells of the mice immunized and stimulated with F1 and PmMSP1_19_ were those with the highest levels of IL-4. IL-10 cytokine is anti-inflammatory, secreted by activated Th2 cells, and is believed to limit harmful inflammatory responses during the blood stage of the parasite in mice [48]. IL-10 was found at a higher level in sera collected from mice immunized with F1 and with PmMSP1_19_. The cytokines produced by each subset promote the polarization, where the cytokines produced by Th1 cells negatively regulate the Th2 response, and vice versa [49]. Therefore, stimulation of Th1/Th2 subsets by immunization with recombinant proteins is important, since homeostasis between Th1/Th2 cells may achieve a balanced regulation between the pro-inflammatory and anti-inflammatory actions in the immune response.

Many studies have shown that both cellular and humoral immunity are involved in protective immunity against malaria [39,50,51]. Both immune responses were triggered by the recombinant proteins derived from PmMSP1. These results suggest that these fragments of *P*. *malariae* may be more widely evaluated as potential vaccine candidates against blood stage parasites. Similar results have been reported in studies involving mice immunized with MSP1_42_ recombinant of *P*. *falciparum* and *P*. *vivax* [36,37].

The affinity of an antibody for its corresponding antigen is an important determinant of the antibody’s biological efficacy [52,53]. For bacteria, it has been shown that affinity of antibodies is important for protection from disease after vaccination [54,55]. More recently, high-affinity antibodies to *P*. *falciparum* merozoite antigens were associated with protection from malaria [56]. Thus, we evaluated the sera from the immunized mice for their affinity against the recombinant proteins (F1, F2, F3, F4, and PmMSP1_19_) in the presence of the chaotropic agent ammonium thiocyanate. Our results showed that the antibodies generated with immunization with PmMSP1_19_ showed the highest affinity, requiring a higher molarity of ammonium thiocyanate to dissociate 50% of the antibodies. In the sera of the animals immunized with the F1, F2, F3 and F4 proteins, the effect of ammonium thiocyanate in the antigen-antibody interaction was very similar. Antibodies with high affinity for the target antigen have also been reported for PvMSP1_19_ [57,58].

### Antigenicity of PmMSP1 recombinant proteins in humans

PmMSP1 recombinant proteins were recognized by IgG antibodies from human sera from patients with malaria caused by *P*. *malariae* in western blot assays suggesting that the presence of the GST did not modify the epitopes of MSP1 recognized by human IgG. GST, originally from *Schistosoma japonicum*, was not recognized by these sera. Our experiments demonstrated that PmMSP1_19_ was antigenic in natural infections, with species-specific recognition. Similar results were also found with human sera from Zimbabwe, Haiti, Cambodia and Mozambique, where species-specific reactivity and rare cross-reactivity of host serum to MSP1_19_ antigens were demonstrated [59,60]. Mice immunized with PvMSP1_19_ and PfMSP1_19_ also showed no reactivity with the respective heterologous antigen [43]. The use of PmMSP1_19_ for detection of antibodies in human malaria patients as well as its serological efficacy for detection of IgG and IgM among experimentally infected chimpanzees has also been demonstrated [61].

Additionally, in our analysis of the reactivity of the F1, F2, F3 and F4 recombinant proteins with sera from positive patients for *P*. *malariae*, *P*. *vivax* and *P*. *falciparum*, we observed that only sera from patients positive for *P*. *malariae* recognized the F1 and F2 proteins. This did not occur for F3 and F4, where the observed ODs were generally lower but sera positive for *P*. *vivax* and *P*. *falciparum* also recognized these proteins. Such cross-reactivity may be a consequence of the higher identity percentage that is observed between these regions and the heterologous proteins (from 50 to 60% at the amino acid level).

By ELISA, we also evaluated recognition of PmMSP1_19_ by serum IgG from Brazilian individuals diagnosed with malaria due to *P*. *malariae* in parallel with sera from patients with other diseases, reaching 100% of sensitivity and specificity. In the particular case of malaria caused by *P*. *vivax*, studies on the naturally acquired humoral immune response against PvMSP1 showed that PvMSP1_19_ is the most immunogenic portion of the molecule and is recognized by 90% of the patients in the region of the Brazilian Amazon with a specificity of 98.3% [62,63]. Studies have also shown that patients from endemic regions have circulating antibodies that specifically recognize the N-terminal region of PvMSP1 [64]. PfMSP1_19_ is also strongly recognized by human sera from endemic regions of malaria [65].

This study represents the first step in assessing humoral and cellular immune responses in animal models, as well as the naturally acquired response to the PmMSP1 antigen. The different recombinant proteins produced here may be useful in sero-epidemiological studies, both in humans as well as non-human primates from different geographic regions, highlighting possible targets in the development of more sensitive and specific diagnostic tests and enabling an evaluation of the exposure to *P*. *malariae* or *P*. *brasilianum* in populations. Since the produced antigens are quite immunogenic, they may also prove useful for the rational design of vaccine formulations against *P*. *malariae* malaria. However, studies as a growth inhibition assay (GIA) and challenges by *P*. *brasilianum* of PmMSP1 protein immunized monkeys are still needed to know the true potential of these antigens as vaccine candidates.

## Supporting information

S1 Fig Analysis of the IgG antibody immune response in each collection, in the mice immunized with the recombinant proteins. F1, F2, F3, F4, PmMSP1_19_ and GST. Antibody titers were detected by ELISA at the 14th, 28th, 45th and 70th days after the first immunization. The results are expressed by geometric mean ± standard deviation pattern. * (p <0.05), ** (p <0.001) and *** (p <0.0001).
(TIF)

S1 File Zip file with Prism files that generate the figures in the paper. (ZIP)
