# Supplementary figures and images for: Recombinant proteins of Plasmodium malariae merozoite surface protein 1 (PmMSP1): Testing immunogenicity in the BALB/c model and potential use as diagnostic tool
Source: PLoS One. 2019 Jul 25;14(7):e0219629. doi: 10.1371/journal.pone.0219629 (PMC6657842; doi:10.1371/journal.pone.0219629)

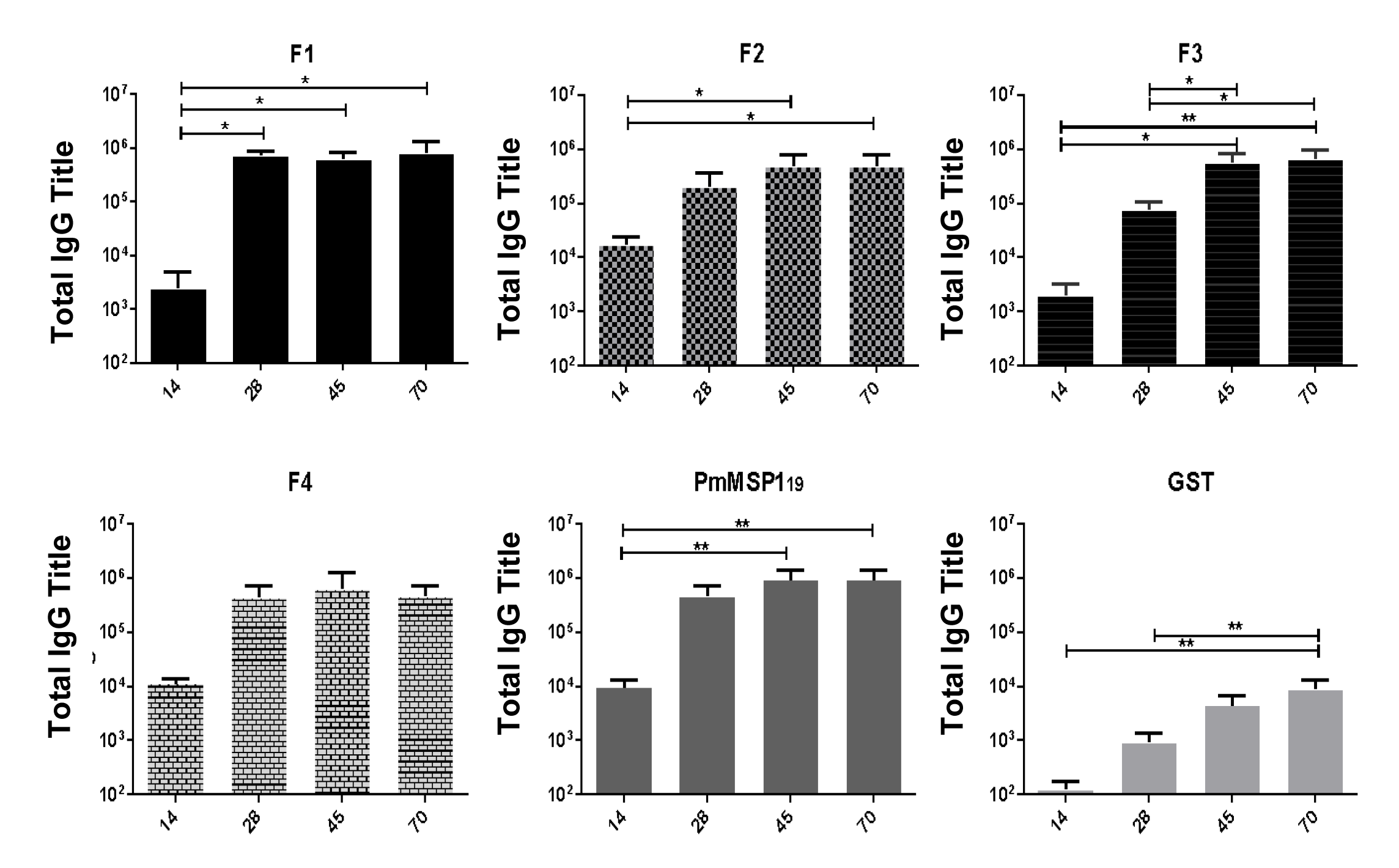

Supplement: S1 Fig — F1, F2, F3, F4, PmMSP119 and GST. Antibody titers were detected by ELISA at the 14th, 28th, 45th and 70th days after the first immunization. The results are expressed by geometric mean ± standard deviation pattern. * (p <0.05), ** (p <0.001) and *** (p <0.0001). (TIF) [file pone.0219629.s001.tif]
